# Disparities in cancer outcomes across age, sex, and race/ethnicity among patients with pancreatic cancer

**DOI:** 10.1002/cam4.1277

**Published:** 2018-01-11

**Authors:** Ryan Nipp, Angela C. Tramontano, Chung Yin Kong, Pari Pandharipande, Emily C. Dowling, Deborah Schrag, Chin Hur

**Affiliations:** ^1^ Department of Medicine Division of Hematology and Oncology Massachusetts General Hospital Cancer Center Boston Massachusetts; ^2^ Harvard Medical School Boston Massachusetts; ^3^ Institute for Technology Assessment Massachusetts General Hospital Boston Massachusetts; ^4^ Dana‐Farber Cancer Institute Harvard Medical School Boston Massachusetts; ^5^ Gastrointestinal Division Harvard Medical School Boston Massachusetts

**Keywords:** Geriatrics, health services, health care disparities, pancreatic cancer, patient outcomes

## Abstract

Age, sex, and racial/ethnic disparities exist, but are understudied in pancreatic adenocarcinoma (PDAC). We used the Surveillance, Epidemiology, and End Results (SEER)–Medicare linked database to determine whether survival and treatment disparities persist after adjusting for demographic and clinical characteristics. Our study included PDAC patients diagnosed between 1992 and 2011. We used Cox regression to compare survival across age, sex, and race/ethnicity within early‐stage and late‐stage cancer subgroups, adjusting for marital status, urban location, socioeconomics, SEER region, comorbidities, stage, lymph node status, tumor location, tumor grade, diagnosis year, and treatment received. We used logistic regression to compare differences in treatment received across age, sex, and race/ethnicity. Among 20,896 patients, 84% were White, 9% Black, 5% Asian, and 2% Hispanic. Median age was 75; 56% were female and 53% had late‐stage cancer. Among early‐stage patients in the adjusted Cox model, older patient subgroups had worse survival compared with ages 66–69 (HR > 1.1, *P* < 0.01 for groups >69); no survival differences existed between sexes. Black (HR = 1.1, *P* = 0.01) and Hispanic (HR = 1.2, *P* < 0.01) patients had worse survival compared with White. Among late‐stage cancer patients, patients over age 84 had worse survival than those aged 66–69 (HR = 1.1, *P* < 0.01), and males (HR = 1.08, *P* < 0.01) had worse survival than females; there were no racial/ethnic differences. Older age and minority race/ethnicity were associated with lower likelihood of receiving chemotherapy, radiation, and/or surgery. Age and racial/ethnic disparities in survival outcomes and treatment received exist for PDAC patients; these disparities persist after adjusting for differences in demographic and clinical characteristics.

## Introduction

Pancreatic cancer is the third leading cause of cancer death in the United States, and estimates suggest it will become the second leading cause of cancer death by 2030 due to an increased number of individuals older than age 65 as the baby boomer generation ages 1. In 2017, over 53,000 new diagnoses of pancreatic cancer are expected in the United States, and projections expect over 43,000 deaths will be attributed to pancreatic cancer this year 2. Incidence rates for this cancer increased at a rate of 1.2% per year between 2000 and 2012 and death rates increased by 0.4% 3. Pancreatic cancer represents a particularly aggressive and lethal malignancy, with approximately 93% of pancreatic cancer patients dying within 5 years of diagnosis 3, 4.

Pancreatic cancer is a disease of aging, occurring at increased rates in older adults, and these patients often experience worse outcomes related to the disease 5, 6, 7. Studies suggest that older patients are typically less likely to receive treatment for their pancreatic cancer and to have worse survival outcomes 8. These poor results may be related to older patients’ complex geriatric issues, such as medical comorbidities, poor nutrition, impaired physical and cognitive function, and limited social support 9, 10, 11, 12, 13. Thus, there is a critical need for further research to characterize the impact of age on treatment received and survival outcomes in patients with pancreatic cancer in order to inform optimal management of the geriatric oncology population.

In addition, prior research suggests that patients’ sex and race/ethnicity may influence the treatment received and survival outcomes among patients with pancreatic cancer. In a study investigating the use and effectiveness of cancer‐directed therapy among patients with locally advanced pancreatic cancer between 1991 and 1996, male patients appeared to be more likely to receive treatment compared with women, yet this finding did not persist after controlling for potential confounders, such as age, place of residence, and comorbidities 14. Research studying racial/ethnic disparities in pancreatic cancer has demonstrated higher incidence rates in the Black population compared with White, Hispanic, and Asian populations 15. Death rates due to pancreatic cancer also appear to be worse in the Black population compared with White, Hispanic, and Asian populations 15. Potential explanations for these survival differences include treatment disparities among different racial and socioeconomic populations 16. Other explanations for survival differences among racial and ethnic groups include factors such as smoking, obesity 17, and tumor characteristics 18.

Although prior studies suggest the presence of age, sex, and racial/ethnic disparities regarding cancer outcomes, additional research is needed to investigate the relationship between these demographic factors and clinical factors such as cancer stage, comorbidity, and treatment received to better understand potential disparities in survival among patients with pancreatic cancer. We used the Surveillance, Epidemiology, and End Results (SEER)–Medicare linked database to study patients with pancreatic adenocarcinoma (PDAC) and further determine whether age, sex, or race/ethnic differences in treatment received and/or survival outcomes exist after controlling for potential confounders such as patient sociodemographics and tumor characteristics. We hypothesized that these potential confounding factors may only partially explain the differences in treatment received and survival among these subgroups, thus highlighting the need for future research to identify additional factors to target in efforts to minimize disparities in pancreatic cancer outcomes.

## Methods

### Cohort inclusion/exclusion criteria

We used data from the 2015 release of the SEER–Medicare database to estimate differences in treatment received and survival among patients with PDAC based on age, sex, and race/ethnicity. The SEER database comprises approximately 28% of the U.S. population and includes information about tumor characteristics, incidence, and survival. The Medicare database includes claims information for approximately 97% of Medicare patients aged 65 or older 19. The SEER–Medicare database links SEER registry data to Medicare enrollment and claims files, including Parts A and B claims for covered health care services, and can be used to examine patterns of care among cancer patients.

Patients were included if they were diagnosed at 65+ and had a microscopically confirmed (histology or cytology) PDAC diagnosed between 1 January 1992 and 31 December 2011. Adenocarcinoma histology was based on the International Classification of Diseases for Oncology (ICD‐O‐3) codes listed in Table S1. Only patients with PDAC as their primary cancer and continuous enrollment in Medicare Parts A and B from 13 months prior to their diagnosis to death or 31 December 2013 were included in our analysis. To ensure that we completely captured claims data regarding heath care services, we excluded patients enrolled in an HMO during this period because no billing information regarding HMO services is available in this database. Stage is based on the derived SEER stage variable for the sixth edition of the AJCC Cancer Staging Manual; those prior to 2004 were classified by mapping the SEER variables for extension of disease and lymph node involvement to the appropriate AJCC sixth edition stage. Patients in which we could not determine AJCC stage were excluded. The N classification variable was based on lymph node involvement (N0, no lymph nodes; N1 at least one node; unknown, node involvement not known). We imputed socioeconomic status by using U.S. Census data provided in SEER–Medicare to derive quintiles of ZIP code‐level median household income. We categorized patients into four race/ethnicity groups (White, Black, Hispanic, Asian) using SEER variables; 62 patients coded as Native American and 58 patients coded as unknown race/ethnicity were excluded. The final cohort was 20,896 patients: 9773 (46.8%) were early stage (I–III) and 11,123 (53.2%) were late stage (stage IV). A flowchart showing how the final cohort was derived is showed in Figure S1.

### Statistical analysis

We compared the distribution of all patient and cancer characteristics included in the analysis across age, sex, and race/ethnic groups using a chi‐square test. We divided patients into two cohorts (early stage [I–III] and late stage [IV]) to examine survival differences across age, sex, and race/ethnicity among early‐stage and late‐stage patients independently. We chose to analyze stage IV separately due to the difference in treatment patterns found in this stage group; metastatic patients are generally offered nonsurgical treatment. We used Kaplan–Meier to estimate survival within subgroups of age, sex, and race/ethnicity, with patients alive on 31 December 2013 censored, and potential differences assessed using the log‐rank test.

We classified patients as having received surgery, radiation, and/or chemotherapy if they had at least one Medicare claim after diagnosis, based on codes listed in Table S1. We calculated comorbidity scores using the Deyo adaptation of the Charlson comorbidity index to Medicare inpatient, outpatient, and physician claims during the 13‐month period prior to cancer diagnosis 20, 21, 22. We classified the scores into three groups (0, 1, and 2+).

We examined whether age, sex, and race/ethnic differences were predictors of treatment receipt (surgery, radiation, or chemotherapy) after controlling for other demographic and clinical predictors using logistic regression models. To examine whether age, sex, and race/ethnic differences in overall survival remained after controlling for other demographic and clinical predictors, we used Cox proportional hazard models in both the early‐stage and late‐stage patient cohorts to compare hazard ratios. We included the following variables in the models: age at diagnosis; sex; race/ethnicity; marital status; urban/rural location; SES (census tract quintile); SEER region, year of diagnosis, AJCC stage, comorbidity score; receipt of surgery, radiation, or chemotherapy; tumor location (pancreatic head, body/tail, or other); N stage (N0, N1, or unknown); and tumor grade (low, intermediate, poor/undifferentiated, or unknown). To assess for interactions between stage (I–III vs. IV) and age, sex, and race/ethnicity, we used Cox proportional hazard models to test for an interaction effect between each of these covariates.

We defined statistical significance as *P* < 0.05 in a two‐sided test. We performed statistical analysis using SAS software, version 9.4 (SAS Institute, Inc., Cary, NC).

## Results

### Demographics and clinical characteristics

In our cohort of 20,896, there were 3906 (19%) patients aged 66–69, 5688 (27%) aged 70–74, 5508 (26%) aged 75–79, 3732 (18%) aged 80–84, and 2062 (10%) aged 85 or older. There were 11,599 (56%) female patients and 9297 (44%) males. Most patients were White (*n* = 17,541; 84%), and fewer were Black (*n* = 1889; 9%), Asian (*n* = 1062; 5%), and Hispanic (*n* = 404; 2%). Patients over age 65 who were excluded (*N* = 30,806) were more likely to be male (47% vs. 44%, *P* < 0.0001), a race/ethnicity other than White (22% vs. 16%, *P* < 0.0001), or diagnosed in the older age subgroup (85+ years old; 12% vs. 10%, *P* < 0.0001). The complete list of demographics and clinical characteristics for the final cohort are found in Tables 1 and 2, respectively.

**Table 1:** Demographics of stage I–IV pancreatic adenocarcinoma patients

Demographic	White	Black	Hispanic	Asian	*P*	66–69	70–74	75–80	80–84	85+	*P*	Male	Female	*P*
Total	17,541 (84%)	1889 (9%)	404 (2%)	1062 (5%)		3906 (19%)	5688 (27%)	5508 (26%)	3732 (18%)	2062 (10%)		9297 (44%)	11,599 (56%)	
Age														
66–69	3232 (18%)	414 (22%)	60 (15%)	200 (19%)	<0.0001									
70–74	4729 (27%)	575 (30%)	94 (23%)	290 (27%)										
75–79	4674 (27%)	432 (23%)	124 (31%)	278 (26%)										
80–84	3167 (18%)	288 (15%)	85 (21%)	192 (18%)										
85+	1739 (10%)	180 (10%)	41 (10%)	102 (10%)										
Sex														
Male	7922 (45%)	687 (36%)	193 (48%)	495 (47%)	<0.0001	2010 (51%)	2722 (48%)	2382 (43%)	1494 (40%)	689 (33%)	<0.0001			
Female	9619 (55%)	1202 (64%)	211 (52%)	567 (53%)		1896 (49%)	2966 (52%)	3126 (57%)	2238 (60%)	1373 (67%)				
Marital status														
Yes	10,183 (58%)	719 (38%)	209 (52%)	669 (63%)	<0.0001	2606 (67%)	3585 (63%)	3089 (56%)	1814 (49%)	686 (33%)	<0.0001	6919 (74%)	4861 (42%)	<0.0001
Urban														
Yes	17,211 (98%)	1875 (99%)	(>97%)^a^	1061 (100%)	<0.0001	3833 (98%)	5595 (98%)	5410 (98%)	3687 (99%)	2026 (98%)	n.s.	9157 (98%)	11,394 (98%)	n.s.
SES^b^														
0 (lowest)	2800 (16%)	981 (52%)	160 (40%)	245 (23%)	<0.0001	800 (21%)	1122 (20%)	1096 (20%)	742 (20%)	426 (21%)	n.s.	1697 (18%)	2489 (21%)	<0.0001
1	3432 (20%)	423 (22%)	102 (25%)	222 (21%)		771 (20%)	1149 (20%)	1116 (20%)	734 (20%)	409 (20%)		1749 (19%)	2430 (21%)	
2	3682 (21%)	225 (12%)	64 (16%)	202 (19%)		763 (20%)	1127 (20%)	1110 (20%)	729 (20%)	444 (22%)		1892 (20%)	2281 (20%)	
3	3785 (22%)	156 (8%)	54 (13%)	186 (18%)		797 (20%)	1128 (20%)	1080 (20%)	759 (20%)	417 (20%)		1876 (20%)	2305 (20%)	
4 (highest)	3842 (22%)	104 (6%)	24 (6%)	207 (19%)		775 (20%)	1162 (20%)	1106 (20%)	768 (21%)	366 (18%)		2083 (22%)	2094 (18%)	
SEER region														
Northeast	4153 (24%)	297 (16%)	49 (12%)	57 (5%)	<0.0001	696 (18%)	1144 (20%)	1330 (24%)	906 (24%)	480 (23%)	<0.0001	1955 (21%)	2601 (22%)	<0.0001
South	3262 (19%)	741 (39%)	a	18 (2%)		852 (22%)	1193 (21%)	1009 (18%)	645 (17%)	330 (16%)		1744 (19%)	2285 (20%)	
Midwest	3219 (18%)	456 (24%)	a	18 (2%)		718 (18%)	1008 (18%)	955 (17%)	668 (18%)	356 (17%)		1598 (17%)	2107 (18%)	
West/Hawaii	6907 (39%)	395 (21%)	335 (83%)	969 (91%)		1640 (42%)	2343 (41%)	2214 (40%)	1513 (41%)	896 (43%)		4000 (43%)	4606 (40%)	
Charlson score^b^														
0	7302 (42%)	616 (33%)	116 (29%)	357 (34%)	<0.0001	1746 (45%)	2356 (41%)	2132 (39%)	1409 (38%)	748 (36%)	<0.0001	3476 (37%)	4915 (42%)	<0.0001
1	5308 (30%)	548 (29%)	155 (38%)	383 (36%)		1211 (31%)	1784 (31%)	1691 (31%)	1091 (29%)	617 (30%)		2859 (31%)	3535 (30%)	
2+	4931 (28%)	725 (38%)	133 (33%)	322 (30%)		949 (24%)	1548 (27%)	1685 (31%)	1232 (33%)	697 (34%)		2962 (32%)	3149 (27%)	

n.s: nonsignificant in chi‐square test.

a Values suppressed in accordance with SEER–Medicare guidelines to mask cell sizes that may be <11 and ensure patient confidentiality. Percentages may not add to 100 due to rounding.

SES: quintiles based on median income by census tract ZIP code.

b Deyo adaptation based on 12 months prior to diagnosis date.

**Table 2:** Cancer characteristics of stage I–IV pancreatic adenocarcinoma patients

Characteristic	White	Black	Hispanic	Asian	*P*	66–69	70–74	75–80	80–84	85+	*P*	Male	Female	*P*
Total	17,541 (84%)	1889 (9%)	404 (2%)	1062 (5%)		3906 (19%)	5688 (27%)	5508 (26%)	3732 (18%)	2062 (10%)		9297 (44%)	11,599 (56%)	
AJCC stage														
I	1309 (7%)	148 (9%)	39 (10%)	81 (8%)	<0.0001	232 (6%)	338 (6%)	406 (7%)	331 (9%)	270 (13%)	<0.0001	648 (7%)	929 (8%)	0.005
II	5585 (32%)	495 (26%)	126 (31%)	349 (33%)		1243 (32%)	1842 (32%)	1742 (32%)	1161 (31%)	567 (28%)		2893 (31%)	3662 (32%)	
III	1354 (8%)	154 (8%)	31 (8%)	102 (10%)		314 (8%)	424 (7%)	453 (8%)	290 (8%)	160 (8%)		707 (8%)	934 (8%)	
IV	9293 (53%)	1092 (58%)	208 (51%)	530 (50%)		2117 (54%)	3084 (54%)	2907 (53%)	1950 (52%)	1065 (52%)		5049 (54%)	6074 (52%)	
Grade														
Low	1007 (6%)	109 (6%)	26 (6%)	56 (5%)	n.s.	216 (6%)	353 (6%)	304 (6%)	221 (6%)	104 (5%)	<0.0001	526 (6%)	672 (6%)	0.004
Intermediate	3359 (19%)	333 (18%)	61 (15%)	226 (21%)		817 (21%)	1162 (20%)	1072 (19%)	634 (17%)	294 (14%)		1708 (18%)	2271 (20%)	
Poor/undifferentiated	3525 (20%)	363 (19%)	89 (22%)	221 (21%)		850 (22%)	1184 (21%)	1118 (20%)	714 (19%)	332 (16%)		1965 (21%)	2233 (19%)	
Unknown	9650 (55%)	1084 (57%)	228 (56%)	559 (53%)		2023 (52%)	2989 (53%)	3014 (55%)	2163 (58%)	1332 (65%)		5098 (55%)	6423 (55%)	
N1														
No	7572 (43%)	885 (47%)	195 (48%)	501 (47%)	0.0009	1621 (42%)	2286 (40%)	2385 (43%)	1756 (47%)	1105 (54%)	<0.0001	3979 (43%)	5174 (45%)	0.03
Yes	5321 (30%)	503 (27%)	105 (26%)	3022 (28%)		1307 (33%)	1862 (33%)	1657 (30%)	1009 (27%)	396 (19%)		2804 (30%)	3427 (30%)	
Unknown	4648 (27%)	501 (27%)	104 (26%)	259 (24%)		978 (25%)	1540 (27%)	1466 (27%)	967 (26%)	561 (27%)		2514 (27%)	2998 (26%)	
Pancreas location														
Head	9249 (53%)	943 (50%)	218 (54%)	523 (49%)	0.0003	2029 (52%)	3003 (53%)	2862 (52%)	1972 (53%)	1067 (52%)	n.s.	4772 (51%)	6161 (53%)	0.01
Tail/body	4061 (23%)	516 (27%)	81 (20%)	283 (27%)		956 (24%)	1332 (23%)	1314 (24%)	890 (24%)	449 (22%)		2283 (25%)	2658 (23%)	
Other	4231 (24%)	430 (23%)	105 (26%)	256 (24%)		921 (24%)	1353 (24%)	1332 (24%)	870 (23%)	546 (26%)		2242 (24%)	2780 (24%)	
Year of diagnosis														
1992–1993	877 (5%)	95 (5%)	a	39 (4%)	<0.0001	224 (6%)	336 (6%)	257 (5%)	131 (4%)	71 (3%)	<0.0001	462 (5%)	557 (5%)	n.s.
1994–1996	1280 (7%)	137 (7%)	a	74 (7%)		334 (9%)	475 (8%)	391 (7%)	200 (5%)	107 (5%)		661 (7%)	846 (7%)	
1997–1999	1174 (7%)	123 (7%)	23 (6%)	77 (7%)		289 (7%)	398 (7%)	370 (7%)	237 (6%)	103 (5%)		592 (6%)	805 (7%)	
2000–2002	2939 (17%)	336 (18%)	68 (17%)	149 (14%)		657 (17%)	1021 (18%)	942 (17%)	578 (15%)	294 (14%)		1567 (17%)	1925 (17%)	
2003–2005	3523 (20%)	423 (22%)	94 (23%)	194 (18%)		766 (20%)	1131 (20%)	1176 (21%)	772 (21%)	389 (19%)		1878 (20%)	2356 (20%)	
2006–2008	3777 (22%)	404 (21%)	80 (20%)	230 (22%)		783 (20%)	1123 (20%)	1192 (22%)	898 (24%)	495 (24%)		1999 (22%)	2492 (21%)	
2009–2011	3971 (23%)	371 (20%)	115 (28%)	299 (28%)		853 (22%)	1204 (21%)	1180 (21%)	916 (25%)	603 (29%)		2138 (23%)	2618 (23%)	
Surgery														
Yes	2652 (15%)	185 (10%)	44 (11%)	150 (14%)	<0.0001	664 (17%)	972 (17%)	846 (15%)	435 (12%)	114 (6%)	<0.0001	1389 (15%)	1642 (14%)	n.s.
Radiation														
Yes	4224 (24%)	387 (20%)	81 (20%)	271 (26%)	0.0007	1234 (32%)	1584 (28%)	1281 (23%)	637 (17%)	227 (11%)	<0.0001	2284 (25%)	2679 (23%)	0.01
Chemotherapy														
Yes	9344 (53%)	800 (42%)	185 (46%)	541 (51%)	<0.0001	2582 (66%)	3432 (60%)	2887 (52%)	1494 (40%)	475 (23%)	<0.0001	5090 (55%)	5780 (50%)	<0.0001

n.s.: nonsignificant in chi‐square test.

a Values suppressed in accordance with SEER–Medicare guidelines to mask cell sizes that may be <11 and ensure patient confidentiality. Percentages may not add to 100 due to rounding.

Patients in the 66–69 and 70–74 age groups were more likely to be married, have a lower comorbidity score, and to be diagnosed with late‐stage disease. Male patients were more likely to have a younger diagnosis age, to be married, and they had a higher comorbidity score than female patients. Black and Hispanic patients were less likely to be married, and they had a lower SES, were diagnosed at a younger age, and had a higher Charlson comorbidity score compared with the Asian and White populations. Black patients were also more likely to have late‐stage PDAC.

### Treatment factors

**Figure 1:**
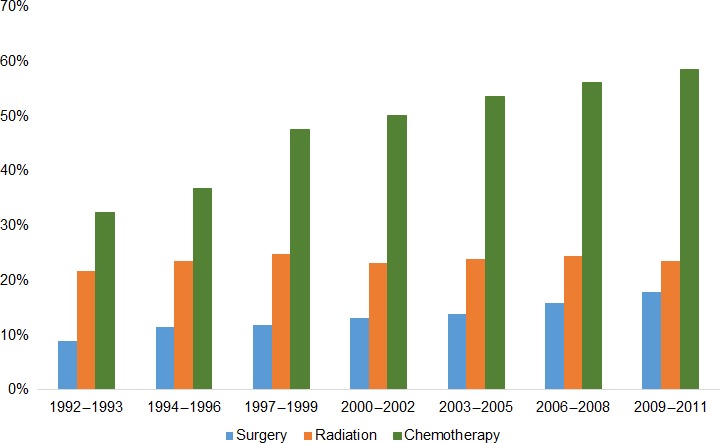
Treatment of stage I–IV pancreatic adenocarcinoma patients by year of diagnosis.

Rates of surgery receipt among the entire cohort increased over time, with 8.9% of patients diagnosed in 1992–1993 receiving surgery and 17.8% diagnosed in 2009–2011 receiving surgery (Fig. 1). Chemotherapy rates also steadily increased, with 32.5% of patients diagnosed in 1992–1993 receiving chemotherapy and 58.5% diagnosed in 2009–2011 receiving chemotherapy. Rates of radiation receipt generally remained stable over time, with 21.7% of patients diagnosed in 1992–1993 receiving radiation and 23.6% diagnosed in 2009–2011 receiving radiation.

Younger patient subgroups (those aged 66–80) were more likely to have received surgery, radiation, or chemotherapy than the older subgroups, and male patients were more likely to have received radiation or chemotherapy than female patients. Black patients were least likely to have received surgery, radiation, or chemotherapy compared to the other racial subgroups. Using logistic regression models, we sought to determine whether the differences in treatment received were significant after adjusting for other baseline sociodemographic and cancer characteristics (full logistic regression modeling results listed in Table S2). For surgery, age (*P* < 0.0001) and race/ethnicity (*P* = 0.0002) and were both significant predictors, while sex was not (*P* = 0.41). These results were consistent for the radiation (age: *P* < 0.0001; sex: *P* = 0.56; race/ethnicity: *P* = 0.001) and chemotherapy (age: *P* < 0.0001; sex: *P* = 0.30; race/ethnicity: *P* = 0.0001) models. Among early‐stage patients, age and race/ethnicity were both significant predictors for surgery (*P* < 0.0001 and *P* = 0.02, respectively) and chemotherapy (*P* < 0.0001 and *P* = 0.004, respectively). Only age (*P* < 0.0001) was a significant predictor of receipt of radiation. Sex was not significant for any of the treatment models. Among late‐stage patients, none of the three variables were significant predictors of surgery, while only age (*P* < 0.0001) was a significant predictor of chemotherapy. Age and race/ethnicity were both significant predictors for radiation (*P* < 0.0001 and *P* = 0.005, respectively), while sex was not significant.

### Survival outcomes

**Figure 2:**
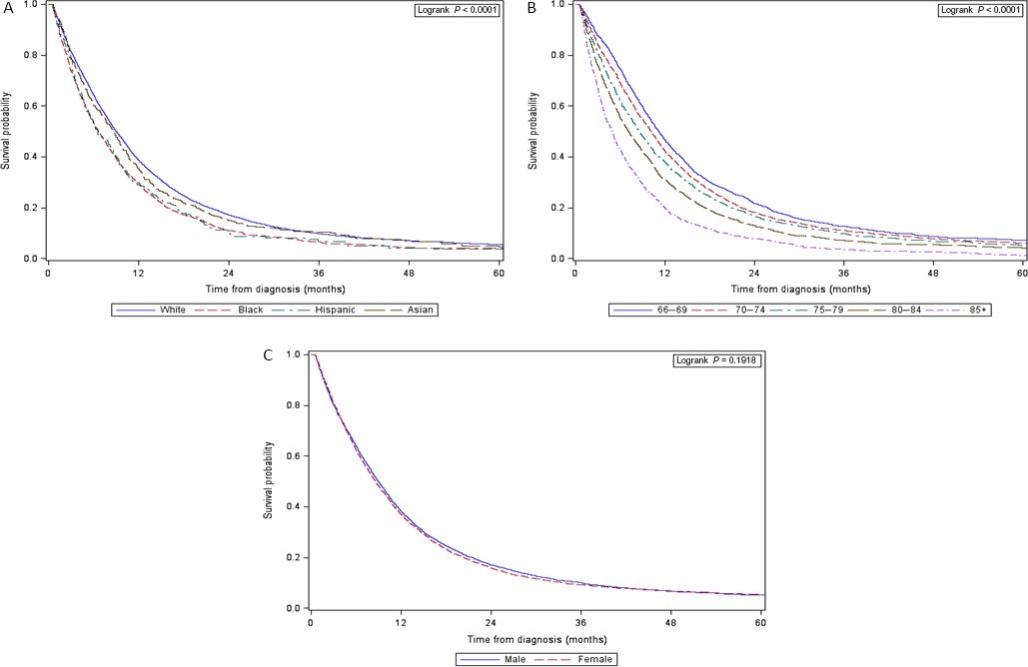
Kaplan–Meier survival curve for early‐stage (I–III) pancreatic adenocarcinoma patients (A) race, (B) age, and (C) sex.

In the univariate Cox proportional hazards model using the interaction terms for stage age, sex, and race/ethnicity, we found significant effects between stage and sex (*P* = 0.005) and stage and race/ethnicity (*P* = 0.0008); however, there was no effect between stage and age (*P* = 0.68). We examined survival outcomes between early‐stage and late‐stage patients separately in both the Kaplan–Meier survival curves and adjusted models. Figure 2 shows the Kaplan–Meier survival curve for early‐stage patients stratified by age, sex, and race/ethnicity. The median overall survival for this cohort was 8.7 months. The median survival decreased among age groups, from 11.2 months for ages 66–69 to 4.8 months for ages 85+. The median survival for males and females was 8.9 and 8.6 months, respectively. Survival was worse for Black (6.6 months) and Hispanic (6.6 months) then for White (9.0 months) and Asian (8.7 months) patients. At 3 years, the survival rates for early‐stage patients were 9.9%, 6.4%, 6.5%, and 10.3% for White, Black, Hispanic, and Asian patients, respectively (survival rates are shown in Table S3).

**Figure 3:**
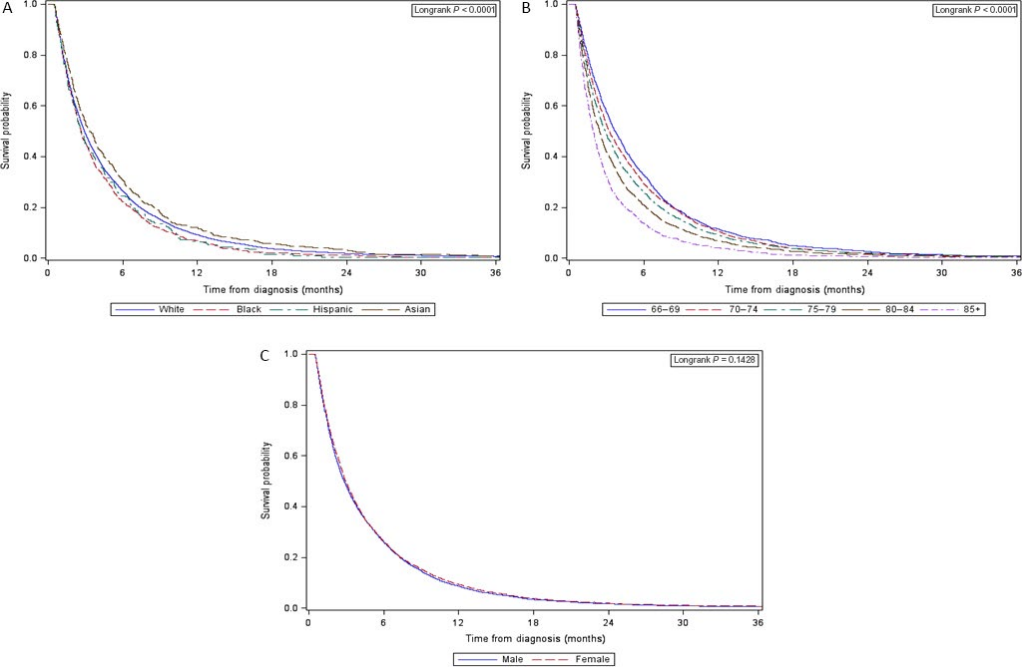
Kaplan–Meier survival curve for late‐stage (IV) pancreatic adenocarcinoma patients (A) race, (B) age, and (C) sex.

Figure 3 displays the Kaplan–Meier survival curves for late‐stage patients stratified by age, sex, and race/ethnicity. The median overall survival for this cohort was 2.9 months. The median survival decreased among age groups, from 5.9 months for ages 66–69 to 3.3 months for ages 85+. The median survival for males and females was 2.8 and 2.9 months, respectively. Survival was worse for Black (2.6 months) and Hispanic (2.6 months) than for White (2.9 months) and Asian (3.3 months) patients. For late‐stage patients, the 2‐year survival rates were 1.9%, 1.1%, 0.9%, and 3.0% for White, Black, Hispanic, and Asian patients, respectively (survival rates are shown in Table S3).

### Adjustment effects

We ran separate Cox proportional hazards models on the early‐stage (I–III) and late‐stage (IV) patients (full models shown in Table S4). Older patients had worse survival when compared to patients aged 66–69 (HR: 1.1 and above for all age groups, *P* < 0.01 for all) in both the unadjusted and adjusted models, but we found no survival differences between sexes (Table 3). In the unadjusted model of early‐stage cancer patients, Black (HR 1.27, *P* < 0.0001) and Hispanic (HR 1.26, *P* = 0.002) patients had worse survival when compared to White patients. These survival differences persisted after adjustment for patient and tumor characteristics (Black [HR 1.11, *P* = 0.01] and Hispanic [HR 1.24, *P* = 0.005]). Asian patients had no survival differences when compared to White patients in both the unadjusted (HR 1.06, *P* = 0.24) and adjusted (HR 1.06, *P* = 0.26) models.

**Table 3:** Cox proportional hazard ratios for overall survival, adjusted for patient characteristics

Unadjusted models				Adjusted model^a^, ^b^		
	HR	95% CI	*P*	HR	95% CI	*P*
Early stage (I–III) cohort						
Race/ethnicity						
White (ref)	1			1		
Black	1.27	1.18–1.37	<0.0001	1.11	1.02–1.20	0.01
Hispanic	1.26	1.09–1.46	0.002	1.24	1.07–1.43	0.005
Asian	1.06	0.97–1.16	0.24	1.06	0.96–1.15	0.26
Age						
66–69 (ref)	1			1		
70–74	1.11	1.04–1.18	0.0009	1.10	1.02–1.16	0.003
75–79	1.23	1.16–1.31	<0.0001	1.12	1.05–1.19	0.0004
80–84	1.44	1.35–1.54	<0.0001	1.19	1.11–1.28	<0.0001
85+	2.00	1.85–2.17	<0.0001	1.37	1.26–1.49	<0.0001
Sex						
Female (ref)	1			1		
Male	0.97	0.93–1.01	0.19	1.04	0.99–1.08	0.12
Late stage (IV) cohort						
Race/ethnicity						
White (ref)	1			1		
Black	1.13	1.06–1.20	0.0002	0.997	0.93–1.07	0.94
Hispanic	1.09	0.95–1.25	0.22	1.03	0.90–1.19	0.65
Asian	0.89	0.81–0.97	0.009	0.82	0.75–0.90	<0.0001
Age						
66–69 (ref)	1			1		
70–74	1.07	1.02–1.14	0.01	1.05	0.999–1.11	0.07
75–79	1.16	1.10–1.23	<0.0001	1.05	0.99–1.11	0.10
80–84	1.34	1.26–1.43	<0.0001	1.06	0.994–1.13	0.08
85+	1.73	1.60–1.86	<0.0001	1.14	1.06–1.24	0.0006
Sex						
Female (ref)	1			1		
Male	1.03	0.99–1.07	0.14	1.08	1.04–1.13	<0.0001

a Variables included in the adjusted model: AJCC cancer stage, N stage, Charlson comorbidity score, year of diagnosis, SEER region, SES, tumor location, surgery, radiation, chemotherapy, tumor grade, marital status, urban location.

b Variables included in the adjusted model: race/ethnicity, age at diagnosis, sex, Charlson comorbidity score, year of diagnosis, SEER region, SES, tumor location, surgery, radiation, chemotherapy, tumor grade, marital status, urban location.

Among late‐stage cancer patients, the oldest patient age group had worse survival when compared to younger patients (HR: 1.1 for age 85+ compared to 66–69 years, *P* < 0.01), and males (HR: 1.08, *P* < 0.01) had worse survival than females. Black and Hispanic patients had no survival difference compared with White patients (Table 3).

## Discussion

Our study demonstrates disparities for treatment received and survival across age, sex, and race/ethnicity among patients with pancreatic cancer. We found that older patients and Black and Hispanic patients with early‐stage pancreatic cancer experience worse survival outcomes compared to their counterparts even after adjusting for potential confounding sociodemographic and clinical factors. We also found that various clinical factors such as comorbidities and treatment do not appear to fully explain the survival differences among the age and race/ethnic subgroups. Among patients with late‐stage disease, the survival differences across age and race/ethnicity were largely insignificant after adjustment. This is potentially due to the fact that these patients tend to present at a very late stage in their disease, with short survival times. Notably, our findings suggest similar outcomes for male and female patients in early‐stage disease, although males had slightly worse survival than females in the adjusted model of late‐stage pancreatic cancer. Collectively, these findings suggest that disparities persist for patients with pancreatic cancer across age, sex, and race/ethnicity, yet ongoing research is needed to further determine the etiology of these disparities.

Our findings align with several previous studies demonstrating survival differences based on patient age and race/ethnicity 16, 23, 24. For example, Amin (2012) demonstrated that both treatment prevalence and survival decreased as age increased, despite any recent advances in these regimens 8. Singal et al. showed that Black patients experience worse survival than White patients, even after controlling for patient and tumor characteristics, yet there were no survival differences between White patients and the Asian or Hispanic population 16. Other studies have shown mixed results regarding outcomes in the Hispanic patient population. Research by Jou et al. demonstrated similar overall survival among Hispanic and Black patients when compared to White patients treated in a New York City cancer center, while Bathe et al. showed that Hispanic patients had worse outcomes compare to White patients after tumor resection in the pancreas head 25, 26.

Our research further adds to this work by providing an updated analysis that included additional important variables. Many of these studies used SEER without linkage to Medicare data, which does not include important treatment factors such as receipt of chemotherapy, and individual patient factors such as medical comorbidities. The use of a linked SEER–Medicare database allowed us to adjust for these vital factors unavailable in SEER, including comorbidities, chemotherapy use, and socioeconomic status based on zip code and median income. In addition, our study includes more recent SEER data, which may be significant as treatments for pancreatic cancer have rapidly evolved in more recent years. Importantly, our findings suggest that even after including this additional factors, such as chemotherapy, comorbidities, and other tumor characteristics, disparities in receipt of treatment do not fully explain the disparities in survival differences across age, sex, and race/ethnicity.

The persistence of these disparities suggests the existence of additional unmeasured factors that we could not control for. In addition, biases and interactions that we could not control for may also play a role in the disparities we demonstrated. For example, differences in tumor biology by both age and race/ethnicity may be an important factor, which needs to be studied in future research. Prior work on other cancers, such as ovarian and prostate, suggests that malignancies detected at an older age are more aggressive, but this needs to be studied within pancreatic cancer 27, 28, 29. Similarly, heterogeneity in tumor indolence/invasiveness has been reported by race/ethnicity for various cancers, such as a 2006 study by Lee et al. demonstrating that Black patients present with bladder cancer at a higher stage and tumor grade 30, 31, 32. Research by Powell et al. suggests that prostate cancer grows more rapidly and becomes more aggressive among Black men when compared to White. These potential differences in other cancers highlight the need for further research in this area in pancreatic cancer patients.

Our study is subject to several limitations inherent to administrative data. Since SEER–Medicare only includes patients 65 years or older and we excluded 65‐year‐old patients to allow us to calculate comorbidity scores in the year prior to diagnosis, we were unable to conduct analyses on younger patient populations. However, pancreatic cancer is more common among older age groups, with approximately 66% of patients aged 65 or older 33. Other factors that could explain the poorer survival outcomes across age, sex, and race/ethnicity, such as smoking history, family history, and obesity are not completely reported in the SEER–Medicare database. For example, BMI category claim codes were recorded for only approximately 200 patients out of our cohort of 20,896. A previous study by Arnold et al. 17 demonstrated that factors such as smoking and obesity play a major role in pancreatic cancer risk, with obesity playing a significant role in risk among White men and women, and among Black men, although this study could not show whether these contributed to the outcome disparities seen among racial groups. Further research into genetic and other factors is needed, with the goal of helping to better explain these disparities.

In summary, age, sex, and racial disparities in survival outcomes and treatment received exist for patients with pancreatic cancer. These disparities persist after adjusting for differences in sociodemographic, clinical, and treatment characteristics. Future research into other potential factors that can help further explain these findings is warranted.

## Conflicts of Interest

There are no conflicts of interest to disclose.

## Supporting information


**Table S1.** Medicare billing and SEER codes.
**Figure S1.** Flowchart of inclusion/exclusion criteria.
**Table S2.** Factors associated with receipt of treatment.
**Table S3.** Kaplan–Meier overall survival rate estimates.
**Table S4.** Cox proportional hazard ratios for overall survival, adjusted for patient characteristics.
