# Supplementary material for: Disparities in cancer outcomes across age, sex, and race/ethnicity among patients with pancreatic cancer
Source: Cancer Med. 2018 Jan 11;7(2):525–35. doi: 10.1002/cam4.1277 (PMC5806100; doi:10.1002/cam4.1277)
Supplement: Supplementary file 1 — Table S1. Medicare billing and SEER codes. Figure S1. Flowchart of inclusion/exclusion criteria. Table S2. Factors associated with receipt of treatment. Table S3. Kaplan–Meier overall survival rate estimates. Table S4. Cox proportional hazard ratios for overall survival, adjusted for patient characteristics. [file CAM4-7-525-s001.docx]

**Appendix Table 1. Medicare Billing and SEER Codes**

| **Variable** | **Codes** |
| --- | --- |
| Histology | ICD-O-3: 8050, 8140-8147, 8160-8162, 8180-8221, 8250-8507, 8514, 8520-8551, 8560, 8570-8574, 8576, 8940-8941 |
| Surgery | ICD-9-CM: 52.6, 52.51, 52.59, 52.53, 52.52, 52.5, 52.7, 52.96  CPT: 48140, 48145, 48146, 48148, 48150-48155 |
| Radiation therapy | ICD-9-CM: V58.0, 92.21-92.29  CPT: 7331-7336, 73399, 77400- 77499, 77750- 77799  Revenue Center: 0330, 0333, 0339 |
| Chemotherapy | ICD-9-CM: V58.1, 99.25  HCPCS: C1166, C1168, C1179, C9110, C9205, C9207, C9213-C9216, C9411, C9414-C9419, C942x, C9430-C9438, G0345-G0363, J9000–J9999, Q0083–Q0085  CPT: 9651x-9654x, 964xx  Revenue Center 0331, 0332, 0335 |

**Appendix Figure 1: Flowchart of Inclusion/Exclusion Criteria**

**
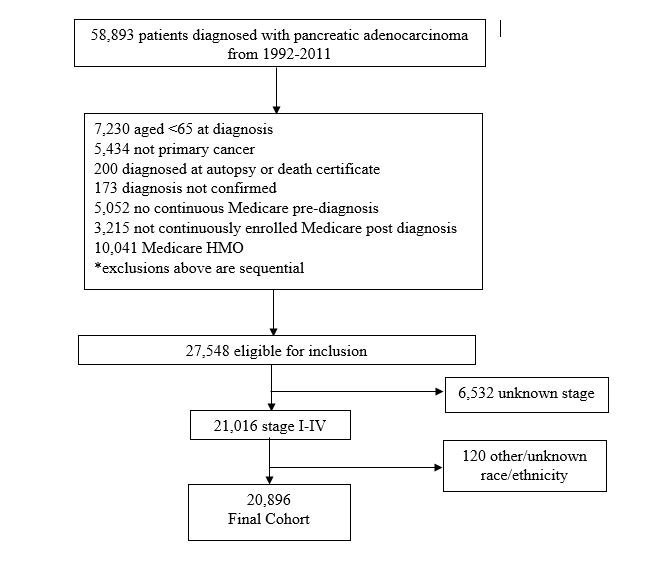
**

**Appendix Table 2: Factors Associated with Receipt of Treatment**

**Surgery**

| **Early Stage(I-III)** | | | | **Late Stage (IV)** | | |
| --- | --- | --- | --- | --- | --- | --- |
|  | **OR** | **95% CI** | **p-value** | **OR** | **95% CI** | **p-value** |
| **Race/Ethnicity** |  |  |  |  |  |  |
| White (ref) | 1 |  |  | 1 |  |  |
| Black | 0.79 | 0.64-0.97 | 0.03 | 0.67 | 0.35-1.28 | 0.22 |
| Hispanic | 0.68 | 0.46-1.02 | 0.06 | 0.65 | 0.16-2.73 | 0.56 |
| Asian | 0.81 | 0.64-1.03 | 0.09 | 0.71 | 0.32-1.56 | 0.39 |
|  |  |  |  |  |  |  |
| **Age** |  |  |  |  |  |  |
| 66-69 (ref) | 1 |  |  | 1 |  |  |
| 70-74 | 1.02 | 0.88-1.19 | 0.77 | 1.29 | 0.84-1.97 | 0.25 |
| 75-79 | 0.92 | 0.79-1.07 | 0.25 | 0.77 | 0.48-1.25 | 0.29 |
| 80-84 | 0.58 | 0.49-0.69 | <0.0001 | 0.96 | 0.57-1.62 | 0.89 |
| 85+ | 0.28 | 0.21-0.35 | <0.0001 | 0.55 | 0.25-1.21 | 0.14 |
|  |  |  |  |  |  |  |
| **Sex** |  |  |  |  |  |  |
| Female (ref) | 1 |  |  | 1 |  |  |
| Male | 1.02 | 0.92-1.14 | 0.69 | 0.88 | 0.64-1.23 | 0.46 |
|  |  |  |  |  |  |  |
| **AJCC Stage** |  |  |  | N/A |  |  |
| I (ref) | 1 |  |  |  |  |  |
| II | 0.74 | 0.63-0.86 | 0.0001 |  |  |  |
| III | 0.14 | 0.11-0.19 | <0.0001 |  |  |  |
|  |  |  |  |  |  |  |
| **N stage** |  |  |  |  |  |  |
| N0 (ref) | 1 |  |  | N/A |  |  |
| N1 | 2.15 | 1.91-2.42 | <0.0001 |  |  |  |
| unk | 0.20 | 0.11-0.39 | <0.0001 |  |  |  |
|  |  |  |  |  |  |  |
| **Charlson Score** |  |  |  |  |  |  |
| 0 (ref) | 1 |  |  | 1 |  |  |
| 1 | 1.02 | 0.91-1.16 | 0.72 | 0.85 | 0.59-1.23 | 0.39 |
| 2+ | 0.83 | 0.73-0.94 | 0.004 | 0.74 | 0.49-1.10 | 0.14 |
|  |  |  |  |  |  |  |
| **Year of Diagnosis** |  |  |  |  |  |  |
| 1992-1993 (ref) | 1 |  |  | 1 |  |  |
| 1994-1996 | 1.39 | 0.995-1.94 | 0.053 | 0.57 | 0.24-1.21 | 0.21 |
| 1997-1999 | 1.48 | 1.06-2.08 | 0.02 | 0.74 | 0.31-1.73 | 0.48 |
| 2000-2002 | 2.00 | 1.48-2.70 | <0.0001 | 1.04 | 0.51-2.13 | 0.91 |
| 2003-2005 | 2.20 | 1.64-2.97 | <0.0001 | 1.59 | 0.80-3.13 | 0.18 |
| 2006-2008 | 3.26 | 2.43-4.40 | <0.0001 | 1.85 | 0.94-3.64 | 0.07 |
| 2009-2011 | 4.04 | 3.01-5.43 | <0.0001 | 1.47 | 0.73-2.96 | 0.28 |
|  |  |  |  |  |  |  |
| **SEER Region** |  |  |  |  |  |  |
| Northeast (ref) | 1 |  |  | 1 |  |  |
| South | 0.64 | 0.54-0.75 | <0.0001 | 1.20 | 0.73-1.99 | 0.47 |
| Midwest | 0.80 | 0.66-0.95 | 0.01 | 1.29 | 0.75-2.21 | 0.37 |
| West/Hawaii | 0.79 | 0.69-0.91 | 0.001 | 1.34 | 0.88-2.06 | 0.17 |
|  |  |  |  |  |  |  |
| **SES** |  |  |  |  |  |  |
| 0 (lowest) (ref) | 1 |  |  | 1 |  |  |
| 1 | 0.95 | 0.80-1.12 | 0.54 | 1.09 | 0.64-1.86 | 0.74 |
| 2 | 0.93 | 0.79-1.11 | 0.42 | 1.32 | 0.79-1.99 | 0.29 |
| 3 | 1.06 | 0.90-1.26 | 0.49 | 1.29 | 0.75-2.21 | 0.41 |
| 4 (highest) | 1.29 | 1.09-1.53 | 0.004 | 1.34 | 0.80-2.25 | 0.27 |

**Surgery (continued)**

| **Pancreas Location** |  |  |  |  |  |  |
| --- | --- | --- | --- | --- | --- | --- |
| Head (ref) | 1 |  |  | 1 |  |  |
| Tail/Body | 1.07 | 0.92-1.23 | 0.39 | 0.98 | 0.69-1.38 | 0.90 |
| Other | 0.81 | 0.68-0.96 | 0.01 | 0.39 | 0.25-0.61 | <0.0001 |
|  |  |  |  |  |  |  |
| **Grade** |  |  |  |  |  |  |
| Low (ref) | 1 |  |  | 1 |  |  |
| Intermediate | 1.37 | 1.15-1.63 | 0.0004 | 1.37 | 0.75-2.49 | 0.30 |
| Poor/Undifferentiated | 1.22 | 1.02-1.46 | 0.03 | 0.90 | 0.49-1.63 | 0.72 |
| Unknown | 0.81 | 0.68-0.96 | <0.0001 | 0.09 | 0.04-0.17 | <0.0001 |
|  |  |  |  |  |  |  |
| **Marital Status** |  |  |  |  |  |  |
| No (ref) | 1 |  |  | 1 |  |  |
| Yes | 1.18 | 1.05-1.32 | 0.006 | 1.31 | 0.91-1.88 | 0.14 |
| Unknown | 1.02 | 0.73-1.40 | 0.93 | 0.93 | 0.28-3.03 | 0.90 |
|  |  |  |  |  |  |  |
| **Urban** |  |  |  |  |  |  |
| No (ref) | 1 |  |  | 1 |  |  |
| Yes | 0.98 | 0.65-1.48 | 0.94 | 2.54 | 0.34-18.76 | 0.36 |

**Radiation**

| **Early Stage(I-III)** | | | | **Late Stage (IV)** | | |
| --- | --- | --- | --- | --- | --- | --- |
|  | **OR** | **95% CI** | **p-value** | **HR** | **95% CI** | **p-value** |
| **Race/Ethnicity** |  |  |  |  |  |  |
| White (ref) | 1 |  |  | 1 |  |  |
| Black | 0.88 | 0.75-1.04 | 0.15 | 0.94 | 0.74-1.19 | 0.58 |
| Hispanic | 0.97 | 0.71-1.34 | 0.86 | 0.97 | 0.58-1.62 | 0.91 |
| Asian | 1.06 | 0.87-1.29 | 0.56 | 1.63 | 1.24-2.14 | 0.0005 |
|  |  |  |  |  |  |  |
| **Age** |  |  |  |  |  |  |
| 66-69 (ref) | 1 |  |  | 1 |  |  |
| 70-74 | 0.76 | 0.67-0.86 | <0.0001 | 0.94 | 0.79-1.11 | 0.45 |
| 75-79 | 0.55 | 0.48-0.62 | <0.0001 | 0.74 | 0.62-0.89 | 0.001 |
| 80-84 | 0.33 | 0.29-0.38 | <0.0001 | 0.58 | 0.47-0.73 | <0.0001 |
| 85+ | 0.18 | 0.15-0.22 | <0.0001 | 0.46 | 0.34-0.62 | <0.0001 |
|  |  |  |  |  |  |  |
| **Sex** |  |  |  |  |  |  |
| Female (ref) | 1 |  |  |  |  |  |
| Male | 0.99 | 0.90-1.08 | 0.75 | 0.97 | 0.84-1.11 | 0.62 |
|  |  |  |  |  |  |  |
| **AJCC Stage** |  |  |  |  |  |  |
| I (ref) | 1 |  |  | N/A |  |  |
| II | 1.12 | 0.98-1.28 | 0.09 |  |  |  |
| III | 1.01 | 0.86-1.18 | 0.94 |  |  |  |
|  |  |  |  |  |  |  |
| **N stage** |  |  |  | N/A |  |  |
| N0 (ref) | 1 |  |  |  |  |  |
| N1 | 1.003 | 0.91-1.11 | 0.95 |  |  |  |
| unk | 0.51 | 0.40-0.65 | <0.0001 |  |  |  |
|  |  |  |  |  |  |  |
| **Charlson Score** |  |  |  |  |  |  |
| 0 (ref) | 1 |  |  | 1 |  |  |
| 1 | 0.85 | 0.77-0.94 | 0.001 | 0.93 | 0.80-1.08 | 0.31 |
| 2+ | 0.65 | 0.58-0.72 | <0.0001 | 0.79 | 0.67-0.93 | 0.004 |
|  |  |  |  |  |  |  |
| **Year of Diagnosis** |  |  |  |  |  |  |
| 1992-1993 (ref) | 1 |  |  | **1** |  |  |
| 1994-1996 | 1.07 | 0.82-1.40 | 0.61 | 1.002 | 0.72-1.39 | 0.99 |
| 1997-1999 | 1.37 | 1.04-1.80 | 0.02 | 1.02 | 0.73-1.42 | 0.93 |
| 2000-2002 | 1.24 | 0.98-1.58 | 0.08 | 1.001 | 0.75-1.35 | 0.99 |
| 2003-2005 | 1.37 | 1.08-1.73 | 0.009 | 0.87 | 0.65-1.18 | 0.37 |
| 2006-2008 | 1.49 | 1.18-1.88 | 0.0008 | 0.97 | 0.72-1.31 | 0.84 |
| 2009-2011 | 1.32 | 1.05-1.67 | 0.02 |  |  | 0.40 |
|  |  |  |  |  |  |  |
| **SEER Region** |  |  |  |  |  |  |
| Northeast (ref) | 1 |  |  | 1 |  |  |
| South | 0.77 | 0.67-0.87 | <0.0001 | 0.76 | 0.610.94 | 0.01 |
| Midwest | 1.19 | 1.04-1.37 | 0.02 | 1.14 | 0.93-1.38 | 0.21 |
| West/Hawaii | 0.66 | 0.58-0.74 | <0.0001 | 0.82 | 0.69-0.98 | 0.03 |
|  |  |  |  |  |  |  |
| **SES** |  |  |  |  |  |  |
| 0 (lowest) (ref) | 1 |  |  | 1 |  |  |
| 1 | 0.99 | 0.86-1.14 | 0.87 | 0.99 | 0.81-1.22 | 0.95 |
| 2 | 1.002 | 0.87-1.15 | 0.98 | 0.97 | 0.78-1.19 | 0.74 |
| 3 | 1.05 | 0.91-1.21 | 0.48 | 0.95 | 0.77-1.18 | 0.64 |
| 4 (highest) | 1.10 | 0.96-1.26 | 0.19 | 1.09 | 0.88-1.34 | 0.43 |

**Radiation (continued)**

| **Pancreas Location** |  |  |  |  |  |  |
| --- | --- | --- | --- | --- | --- | --- |
| Head (ref) | 1 |  |  | 1 |  |  |
| Tail/Body | 1.01 | 0.90-1.13 | 0.90 | 0.78 | 0.67-0.91 | 0.002 |
| Other | 0.91 | 0.80-1.04 | 0.17 | 0.70 | 0.60-0.82 | <0.0001 |
|  |  |  |  |  |  |  |
| **Grade** |  |  |  |  |  |  |
| Low (ref) | 1 |  |  | 1 |  |  |
| Intermediate | 1.15 | 0.97-1.36 | 0.11 | 0.75 | 0.53-1.04 | 0.09 |
| Poor/Undifferentiated | 1.09 | 0.92-1.30 | 0.31 | 0.75 | 0.55-1.04 | 0.08 |
| Unknown | 1.08 | 0.91-1.27 | 0.38 | 0.58 | 0.43-0.79 | 0.0005 |
|  |  |  |  |  |  |  |
| **Marital Status** |  |  |  |  |  |  |
| No (ref) | 1 |  |  | 1 |  |  |
| Yes | 1.27 | 1.15-1.39 | <0.0001 | 1.20 | 1.04-1.39 | 0.01 |
| Unknown | 0.94 | 0.72-1.23 | 0.63 | 1.08 | 0.71-1.65 | 0.71 |
|  |  |  |  |  |  |  |
| **Urban** |  |  |  |  |  |  |
| No (ref) | 1 |  |  | 1 |  |  |
| Yes | 1.36 | 0.97-1.91 | 0.08 | 1.09 | 0.71-1.65 | 0.60 |

**Chemotherapy**

| **Early Stage(I-III)** | | | | **Late Stage (IV)** | | |
| --- | --- | --- | --- | --- | --- | --- |
|  | **OR** | **95% CI** | **p-value** | **OR** | **95% CI** | **p-value** |
| **Race/Ethnicity** |  |  |  |  |  |  |
| White (ref) | 1 |  |  | 1 |  |  |
| Black | 0.75 | 0.64-0.89 | 0.0008 | 0.84 | 0.73-0.98 | 0.02 |
| Hispanic | 0.81 | 0.59-1.10 | 0.18 | 0.89 | 0.66-1.20 | 0.44 |
| Asian | 0.88 | 0.73-1.08 | 0.22 | 0.91 | 0.75-1.11 | 0.35 |
|  |  |  |  |  |  |  |
| **Age** |  |  |  |  |  |  |
| 66-69 (ref) | 1 |  |  | 1 |  |  |
| 70-74 | 0.69 | 0.60-0.79 | <0.0001 | 0.86 | 0.77-0.97 | 0.01 |
| 75-79 | 0.48 | 0.42-0.55 | <0.0001 | 0.61 | 0.54-0.68 | <0.0001 |
| 80-84 | 0.28 | 0.24-0.32 | <0.0001 | 0.36 | 0.31-0.41 | <0.0001 |
| 85+ | 0.12 | 0.10-0.14 | <0.0001 | 0.18 | 0.15-0.21 | <0.0001 |
|  |  |  |  |  |  |  |
| **Sex** |  |  |  |  |  |  |
| Female (ref) | 1 |  |  |  |  |  |
| Male | 1.03 | 0.94-1.13 | 0.51 | 1.02 | 0.94-1.11 | 0.66 |
|  |  |  |  |  |  |  |
| **AJCC Stage** |  |  |  |  |  |  |
| I | 1 |  |  | N/A |  |  |
| II | 1.30 | 1.14-1.49 | <0.0001 |  |  |  |
| III | 1.38 | 1.17-1.63 | 0.0001 |  |  |  |
|  |  |  |  |  |  |  |
| **N stage** |  |  |  |  |  |  |
| N0 (ref) | 1 |  |  | N/A |  |  |
| N1 | 1.14 | 1.03-1.26 | 0.01 |  |  |  |
| unk | 0.80 | 0.64-0.996 | 0.046 |  |  |  |
|  |  |  |  |  |  |  |
| **Charlson Score** |  |  |  |  |  |  |
| 0 (ref) | 1 |  |  | 1 |  |  |
| 1 | 0.93 | 0.84-1.04 | 0.19 | 0.89 | 0.80-0.97 | 0.01 |
| 2+ | 0.57 | 0.51-0.63 | <0.0001 | 0.57 | 0.52-0.63 | <0.0001 |
|  |  |  |  |  |  |  |
| **Year of Diagnosis** |  |  |  |  |  |  |
| 1992-1993 (ref) | 1 |  |  | 1 |  |  |
| 1994-1996 | 1.24 | 0.95-1.63 | 0.11 | 1.18 | 0.93-1.49 | 0.18 |
| 1997-1999 | 2.10 | 1.59-2.76 | <0.0001 | 2.05 | 1.63-2.59 | <0.0001 |
| 2000-2002 | 2.31 | 1.82-2.94 | <0.0001 | 2.43 | 1.98-2.99 | <0.0001 |
| 2003-2005 | 2.75 | 2.17-3.49 | <0.0001 | 2.86 | 2.33-3.52 | <0.0001 |
| 2006-2008 | 3.58 | 2.82-4.54 | <0.0001 | 3.19 | 2.60-3.92 | <0.0001 |
| 2009-2011 | 4.25 | 3.35-5.39 | <0.0001 | 3.38 | 2.75-4.15 | <0.0001 |
|  |  |  |  |  |  |  |
| **SEER Region** |  |  |  |  |  |  |
| Northeast (ref) | 1 |  | <0.0001 | 1 |  |  |
| South | 0.57 | 0.50-0.65 | <0.0001 | 0.64 | 0.57-0.73 | <0.0001 |
| Midwest | 0.71 | 0.61-0.82 | <0.0001 | 0.65 | 0.57-0.74 | <0.0001 |
| West/Hawaii | 0.67 | 0.60-0.76 | <0.0001 | 0.69 | 0.62-0.77 | <0.0001 |
|  |  |  |  |  |  | <0.0001 |
| **SES** |  |  |  |  |  |  |
| 0 (lowest) (ref) | 1 |  |  | 1 |  |  |
| 1 | 1.08 | 0.94-1.25 | 0.26 | 1.13 | 0.997-1.28 | 0.06 |
| 2 | 1.09 | 0.95-1.26 | 0.22 | 1.18 | 1.04-1.34 | 0.01 |
| 3 | 1.24 | 1.07-1.43 | 0.004 | 1.36 | 1.19-1.54 | <0.0001 |
| 4 (highest) | 1.43 | 1.24-1.65 | <0.0001 | 1.62 | 1.42-1.85 | <0.0001 |

**Chemotherapy (continued)**

| **Pancreas Location** |  |  |  |  |  |  |
| --- | --- | --- | --- | --- | --- | --- |
| Head (ref) | 1 |  |  | 1 |  |  |
| Tail/Body | 1.28 | 1.14-1.45 | <0.0001 | 1.16 | 1.05-1.28 | 0.004 |
| Other | 0.90 | 0.79-1.03 | 0.12 | 0.956 | 0.87-1.05 | 0.36 |
|  |  |  |  |  |  |  |
| **Grade** |  |  |  |  |  |  |
| Low (ref) | 1 |  |  | 1 |  |  |
| Intermediate | 1.13 | 0.95-1.34 | 0.16 | 0.93 | 0.73-1.19 | 0.57 |
| Poor/Undifferentiated | 1.14 | 0.95-1.35 | 0.15 | 0.84 | 0.66-1.06 | 0.14 |
| Unknown | 1.02 | 0.87-1.21 | 0.79 | 0.88 | 0.71-1.10 | 0.28 |
|  |  |  |  |  |  |  |
| **Marital Status** |  |  |  |  |  |  |
| No (ref) | 1 |  |  | 1 |  |  |
| Yes | 1.61 | 1.45-1.77 | <0.0001 | 1.49 | 1.37-1.63 | <0.0001 |
| Unknown | 0.87 | 0.67-1.12 | 0.29 | 1.16 | 0.91-1.49 | 0.22 |
|  |  |  |  |  |  |  |
| **Urban** |  |  |  |  |  |  |
| Yes (ref) | 1 |  |  | 1 |  |  |
| No | 1.30 | 0.93-1.84 | 0.13 | 0.94 | 0.68-1.29 | 0.69 |

**Appendix Table 3: Table: Kaplan Meier Overall Survival Rate Estimates**

| **Early Stage** | **White** | **Black** | **Hispanic** | **Asian** |
| --- | --- | --- | --- | --- |
| 1-year | 38.5% | 29.5% | 28.6% | 35.3% |
| 3-years | 9.9% | 6.4% | 6.5% | 10.3% |
| 5-years | 5.4% | 3.6% | 4.1% | 4.5% |
| **Late Stage** | **White** | **Black** | **Hispanic** | **Asian** |
| 1-year | 9.1% | 6.9% | 6.3% | 11.7% |
| 2-years | 1.9% | 1.1% | 0.5% | 3.0% |
| 3-years | 0.7% | 0.2% | 0% | 1.0% |

| **EarlyStage** | **Male** | **Female** |
| --- | --- | --- |
| 1-year | 38.3% | 37.0% |
| 3-years | 10.0% | 9.2% |
| 5-years | 5.1% | 5.3% |
| **Late Stage** | **Male** | **Female** |
| 1-year | 8.6% | 9.3% |
| 2-years | 1.7% | 1.9% |
| 3-years | 0.7% | 0.7% |

| **EarlyStage** | **66-69** | **70-74** | **75-79** | **80-84** | **85+** |
| --- | --- | --- | --- | --- | --- |
| 1-year | 46.5% | 42.3% | 37.9% | 30.9% | 20.0% |
| 3-years | 12.6% | 11.1% | 9.9% | 7.1% | 3.6% |
| 5-years | 7.3% | 6.1% | 5.1% | 4.2% | 1.2% |
| **Late Stage** | **66-69** | **70-74** | **75-79** | **80-84** | **85+** |
| 1-year | 11.4% | 10.5% | 9.0% | 6.7% | 3.9% |
| 2-years | 2.7% | 1.8% | 1.9% | 1.4% | 0.7% |
| 3-years | 0.7% | 0.7% | 0.8% | 0.6% | 0.2% |

**Appendix Table 4: Cox Proportional Hazard Ratios for Overall Survival, Adjusted for Patient Characteristics**

| **Early Stage (I-III)** | | | | **Late Stage (IV)** | | |
| --- | --- | --- | --- | --- | --- | --- |
|  | **HR** | **95% CI** | **p-value** | **HR** | **95% CI** | **p-value** |
| **Race/Ethnicity** |  |  |  |  |  |  |
| White (ref) | 1 |  |  | 1 |  |  |
| Black | 1.11 | 1.02-1.20 | 0.01 | 0.997 | 0.93-1.07 | 0.94 |
| Hispanic | 1.24 | 1.07-1.43 | 0.005 | 1.03 | 0.90-1.19 | 0.65 |
| Asian | 1.06 | 0.96-1.15 | 0.26 | 0.82 | 0.75-0.90 | <0.0001 |
|  |  |  |  |  |  |  |
| **Age** |  |  |  |  |  |  |
| 66-69 (ref) | 1 |  |  | 1 |  |  |
| 70-74 | 1.10 | 1.02-1.16 | 0.003 | 1.05 | 0.999-1.11 | 0.07 |
| 75-79 | 1.12 | 1.05-1.19 | 0.0004 | 1.05 | 0.99-1.11 | 0.10 |
| 80-84 | 1.19 | 1.11-1.28 | <0.0001 | 1.06 | 0.994-1.13 | 0.08 |
| 85+ | 1.37 | 1.26-1.49 | <0.0001 | 1.14 | 1.06-1.24 | 0.0006 |
|  |  |  |  |  |  |  |
| **Sex** |  |  |  |  |  |  |
| Female (ref) | 1 |  |  | 1 |  |  |
| Male | 1.04 | 0.99-1.08 | 0.12 | 1.08 | 1.04-1.13 | <0.0001 |
|  |  |  |  |  |  |  |
| **AJCC Stage** |  |  |  |  |  |  |
| I (ref) | 1 |  |  | N/A |  |  |
| II | 1.33 | 1.25-1.41 | <0.0001 |  |  |  |
| III | 1.70 | 1.57-1.84 | <0.0001 |  |  |  |
|  |  |  |  |  |  |  |
| **N stage** |  |  |  | N/A |  |  |
| N0 (ref) | 1 |  |  |  |  |  |
| N1 | 1.11 | 1.06-1.16 | <0.0001 |  |  |  |
| unk | 1.53 | 1.38-1.69 | <0.0001 |  |  |  |
|  |  |  |  |  |  |  |
| **Charlson Score** |  |  |  |  |  |  |
| 0 (ref) | 1 |  |  | 1 |  |  |
| 1 | 1.10 | 1.05-1.15 | 0.0004 | 1.09 | 1.04-1.14 | 0.0002 |
| 2+ | 1.29 | 1.23-1.36 | <0.0001 | 1.26 | 1.20-1.31 | <0.0001 |
|  |  |  |  |  |  |  |
| **Year of Diagnosis** |  |  |  |  |  |  |
| 1992-1993 (ref) | 1 |  |  | 1 |  |  |
| 1994-1996 | 1.23 | 1.09-1.39 | 0.001 | 1.04 | 0.94-1.15 | 0.49 |
| 1997-1999 | 1.14 | 1.006-1.30 | 0.04 | 1.10 | 0.998-1.22 | 0.07 |
| 2000-2002 | 1.11 | 0.99-1.23 | 0.07 | 1.07 | 0.98-1.18 | 0.15 |
| 2003-2005 | 1.07 | 0.97-1.20 | 0.07 | 0.99 | 0.91-1.09 | 0.82 |
| 2006-2008 | 0.96 | 0.86-1.07 | 0.18 | 0.996 | 0.91-1.09 | 0.93 |
| 2009-2011 | 0.85 | 0.76-0.96 | 0.04 | 0.93 | 0.86-1.03 | 0.15 |
|  |  |  |  |  |  |  |
| **SEER Region** |  |  |  |  |  |  |
| Northeast (ref) | 1 |  |  | 1 |  |  |
| South | 1.19 | 1.12-1.27 | <0.0001 | 1.08 | 1.02-1.15 | 0.01 |
| Midwest | 1.06 | 0.99-1.13 | 0.10 | 0.96 | 0.90-1.02 | 0.15 |
| West/Hawaii | 1.01 | 0.96-1.07 | 0.62 | 0.97 | 0.92-1.02 | 0.22 |
|  |  |  |  |  |  |  |
| **SES** |  |  |  |  |  |  |
| 0 (lowest) (ref) | 1 |  |  | 1 |  |  |
| 1 | 1.003 | 0.94-1.07 | 0.92 | 1.06 | 0.998-1.12 | 0.06 |
| 2 | 1.005 | 0.94-1.07 | 0.89 | 1.01 | 0.95-1.08 | 0.72 |
| 3 | 0.94 | 0.88-1.00 | 0.049 | 0.96 | 0.90-1.02 | 0.20 |
| 4 (highest) | 0.93 | 0.87-0.99 | 0.03 | 0.997 | 0.94-1.06 | 0.93 |

**Cox Proportional Hazard Ratios for Overall Survival, Adjusted for Patient Characteristics (cont’d)**

| **Pancreas Location** |  |  |  |  |  |  |
| --- | --- | --- | --- | --- | --- | --- |
| Head (ref) | 1 |  |  | 1 |  |  |
| Tail/Body | 0.98 | 0.93-1.04 | 0.47 | 1.13 | 1.08-1.18 | <0.0001 |
| Other | 1.08 | 1.01-1.15 | 0.02 | 1.12 | 1.07-1.18 | <0.0001 |
|  |  |  |  |  |  |  |
| **Surgery** |  |  |  |  |  |  |
| No (ref) | 1 |  |  | 1 |  |  |
| Yes | 0.54 | 0.51-0.56 | <0.0001 | 0.44 | 0.38-0.52 | <0.0001 |
|  |  |  |  |  |  |  |
| **Radiation** |  |  |  |  |  |  |
| No (ref) | 1 |  |  | 1 |  |  |
| Yes | 0.77 | 0.74-0.81 | <0.0001 | 0.71 | 0.66-0.75 | <0.0001 |
|  |  |  |  |  |  |  |
| **Chemotherapy** |  |  |  |  |  |  |
| No (ref) | 1 |  |  | 1 |  |  |
| Yes | 0.57 | 0.54-0.60 | <0.0001 | 0.36 | 0.34-0.37 | <0.0001 |
|  |  |  |  |  |  |  |
| **Grade** |  |  |  |  |  |  |
| Low (ref) | 1 |  |  | 1 |  |  |
| Intermediate | 1.26 | 1.16-1.37 | <0.0001 | 1.38 | 1.23-1.54 | <0.0001 |
| Poor/Undifferentiated | 1.84 | 1.69-2.00 | <0.0001 | 1.67 | 1.49-1.86 | <0.0001 |
| Unknown | 1.68 | 1.55-1.82 | <0.0001 | 1.52 | 1.37-1.68 | <0.0001 |
|  |  |  |  |  |  |  |
| **Marital Status** |  |  |  |  |  |  |
| No (ref) | 1 |  |  | 1 |  |  |
| Yes | 0.94 | 0.90-0.99 | 0.01 | 1.02 | 0.98-1.07 | 0.31 |
| Unknown | 0.95 | 0.84-1.07 | 0.40 | 1.04 | 0.92-1.16 | 0.56 |
|  |  |  |  |  |  |  |
| **Urban** |  |  |  |  |  |  |
| No (ref) | 1 |  |  | 1 |  |  |
| Yes | 0.90 | 0.77-1.06 | 0.20 | 1.14 | 0.98-1.32 | 0.10 |
